# Deposition, microstructure and nanoindentation of multilayer Zr nitride and carbonitride nanostructured coatings

**DOI:** 10.1038/s41598-022-09449-6

**Published:** 2022-04-04

**Authors:** Anwar Ul-Hamid

**Affiliations:** Core Research Facilities, King Fahd University of Petroleum & Minerals, Dhahran, 31261 Saudi Arabia

**Keywords:** Materials science, Nanoscience and technology

## Abstract

Nitrides, carbides, and carbonitrides of transition metal elements like Zr, W, Ti, etc. are generally employed to produce hard coatings. Zirconium-based hard coatings have shown useful applications in the areas of tribology, biomedicine and electrical due to their high thermal stability, hardness, biocompatibility, good erosion, wear, and corrosion resistance. In this study, we created homogeneous and tenacious nanostructured hard coatings based on Zr with good mechanical properties. The magnetron sputter deposition technique was utilized to coat stainless steel 316L substrates with multilayers of Zr/ZrN and ZrN/ZrCN with individual layer thicknesses of 250 and 500 nm for each coating composition. The deposition conditions were adjusted to create two different coating thicknesses of 2 and 3 µm. The thickness of the coating was confirmed using Calotest and the coatings’ morphology and elemental composition were determined utilizing the atomic force microscope and scanning electron microscope equipped with energy dispersive x-ray spectrometer. Coating thickness and adhesion were measured using cross-sectional samples and XRD was utilized to analyze the coatings structure. Nanoindenter was employed to determine the instrumental nanoindentation hardness and elastic modulus. The influence of coating thickness on tribological behavior was further investigated using the ratio of nanohardness-to-elastic modulus (H/E). No evidence of decohesion was observed at the substrate/coatings interface, and the grains of all the coatings were observed to show columnar growth which were homogeneous, compact and dense. The grains of the ZrN/ZrCN coatings were observed to be denser, finer and more compact compared to those of the Zr/ZrN coatings. Correspondingly, higher hardness, modulus and H/E values were exhibited by ZrN/ZrCN than Zr/ZrN coatings. This suggests that the ZrN/ZrCN coatings are capable of exhibiting better wear resistance and fracture toughness. The coatings developed in this investigation are anticipated to be suitable for applications in tribology due to their excellent hardness and H/E properties.

## Introduction

Biomedical implants, household products, gas turbine engine components, and drilling and cutting equipment, are products used in industries such as the oil & gas, power, manufacturing, defense, and medicine, and are normally coated with wear resistant hard coatings in order to extend their service lifetimes. Transition metal carbides, nitrides, and carbonitrides based on Ti, Zr, W, and others, are commonly used in developing hard coatings. Because of their low vapour pressure & high melting point, coatings that are based on zirconium (Zr) are difficult to layer, and they are vulnerable to getting contaminated by carbon and oxygen during the process of deposition. This is presumably the reason for the paucity of studies in the literature on coatings based on Zr. Because of the bonding nature and structure of Zr, thin films based on Zr have high microhardness, excellent biocompatibility, high thermal stability, and excellent wear, erosion, corrosion & oxidation resistance. As a result, they are perfect for applications in tribology, biomedical, corrosion-resistant, electrical, nuclear fuel and decorative applications. Several aspects of ZrN coatings have been studied for a variety of applications. ZrN has been used in tribological applications for microhardness, adhesion and wear resistance, as well as integrated circuits barrier materials^1–22^, anti-erosion materials for turbines & compressor blades^23–25^, electrical & optical properties^26,27^, biocompatible materials^28^, compatible materials for nuclear fuel elements^29–31^, microstructure, composition, anti-corrosion and orientation-dependent materials^32–40^. Researches on the effect of synthesis factors on coating characteristics have also been reported^41–46^. The number of studies on ZrCN coatings in the literature are limited. Among the properties and applications explored for ZrCN are morphology, crystallinity & composition^47^, oxidation and corrosion resistance^48,49^, tribological^50^, synthetic parameters^51^, , biomedical^52–59^, characterization^60^ and optical^61^. The hardness of a given coating is affected by the method of synthesis technique, processing, elemental, coating structure and elemental composition. Because of the influence of the underlying substrate, measuring the hardness of thin coatings is difficult. Nanoindentation is a technique for evaluating thin coatings with small indenters under low stress and penetration depths of tens to hundreds of nanometers. Without perceiving or quantifying the size of the indent, the mechanical properties of the coatings can be determined. The values of elastic modulus and nanoindentation hardness are extracted using analytical models. The microstructures, properties and characterization of single-layer Zr, ZrN and ZrCN deposited by magnetron sputtering have been reported, and ZrN was said to exhibit the highest elastic modulus and hardness while Zr exhibited the least^61^.

Multilayer coatings, especially the ones composed of sub-layers of thicknesses in the nanometer range have been reported to show improved properties over the single layer ones^62–69^. The properties and microstructure of a multilayer Zr/CrN coatings grown by the multi-arc ion plating method have been reported^70^. Braic et al.^71^, developed a super lattice TiAlN/TiAlZrN multilayer coatings of different bilayer periods deposited by cathodic arc method and investigated their performances as wear resistant coatings. The tribological, mechanical, microstructural and microchemical properties of TiN/ZrN multilayer coatings with different bilayer periods and thicknesses have been reported^72^. Recently, many laser-assisted manufacturing techniques for producing multilayer coatings have been used with greater flexibility of control^73,74^. Multilayer nitride coatings of TiN/TiAlN and Ti/TiN/TiAlN have been investigated for biomedical applications^75^. Because of its cost effectiveness and reproducibility, the magnetron sputter deposition technology is widely used in industry^76^. It generates adherent and uniform coatings and uses low deposition temperature, which allows for the creation of coatings with improved properties.

Using the magnetron sputter deposition process, multilayer coatings based on Zr/ZrN and ZrN/ZrCN each of layer thicknesses 250 and 500 nm and coatings thicknesses of 2 and 3 µm were synthesized in this study. SEM equipped with energy dispersive x-ray spectroscopy, atomic force microscopy, and x-ray diffraction were employed to investigate the surface morphology and structure of the coatings. The nanoindentation method was used to measure the mechanical properties; modulus and nanoindentation hardness. The indentation hardness was determined as a function of normal load using the load displacement curve. The effects of the coating thickness and adding of carbon on the coatings properties were also investigated.

## Materials and methods

### Coating synthesis

Magnetron sputter deposition was used to develop multilayer Zr/ZrN and ZrN/ZrCN coatings (total thickness 2 and 3 µm) on (100) Si wafers and stainless steel SS316L substrates (of 16 mm diameter disc, thickness 3 mm). The coatings' cross-sectional fracture morphology was next studied from broken coated Si wafers. The stainless steel substrates were polished with emery paper (ranging from 240 to 2400 grit) before being mirror-polished with a diamond suspension prior to deposition. The samples were cleaned ultrasonically using distilled water, ethanol, and acetone for ten minutes. Following that, in-situ sputter etching was carried out for 900 s at Ar pressure of 1.32 Pa and a steady current of 480 mA. As described by Silva et al.^77^, unbalanced dual magnetron sputtering was used to create the coatings. Two highly pure (99.2%) 200 × 100 mm^2^ Zr targets (rectangular in shape) mounted on unbalanced type-2 magnetrons were used to deposit the coatings in a reactive mode in an atmosphere of Ar/N_2_/C_2_H_2_. For compositional and morphological characterization, the films were deposited onto (100) silicon wafers, as well as onto 316L stainless steel discs with a diameter of 3 mm. As previously described^78^, the substrates were mounted in a matrix-like formation on a rotatable substrate holder. The parameters for creating multilayer Zr/ZrN and ZrN/ZrCN were chosen on the basis of each coating’s deposition rates, adhesion properties, and composition. During the depositions, the bias voltage, chamber temperature and argon flux were respectively kept constant at −50 V, 100 °C and 60 sccm, while the current density of Zr was set between 1.8 and 2.0 A/cm^2^. Table 1 shows the values for the target’s potential, working & base pressures with some other deposition parameters used to deposit the final coatings.

**Table 1:** Films deposition conditions.

Sample ID	Coating composition	Ø C_2_H_2_ (sccm)	Ø N_2_ (sccm)	Time (s)	Base pressure (1 × 10^–4^ Pa)	Working pressure (Pa)	Zr Target-1 potential (V)	Zr Target-2 potential (V)	Substrate current (mA)
PN7	Zr ZrN	0	0 22	2940	3.9	0.49 0.57	298 341	341 400	242
PN8	Zr ZrN	0	0 22	4410	4.0	0.49 0.57	298 341	341 396	240
PN9	Zr ZrN	0	0 22	2940	4.0	0.49 0.57	298 333	337 380	270
PN10	Zr ZrN	0	0 22	4410	4.0	0.49 0.56	398 333	337 388	240
PN11	ZrN ZrCN	0 16	22 12	3024	4.7	0.53 0.55	349 361	404 451	280
PN12	ZrN ZrCN	0 16	22 12	4536	6.5	0.54 0.55	337 357	380 427	230
PN13	ZrN ZrCN	0 16	22 12	3024	4.0	0.53 0.55	337 361	384 435	230
PN14	ZrN ZrCN	0 16	22 12	4536	5.3	0.53 0.55	333 353	388 427	240

### Materials characterization

The surface morphology of coatings was investigated by undertaking high resolution secondary electron imaging of the coatings in their as-received state using field emission scanning electron microscope (FESEM, model TESCAN LYRA 3) equipped with an energy dispersive x-ray spectrometer from Oxford Inc. at an accelerating voltage of 20 kV. The backscattered electron images (BEI) and EDS spectra were collected using JEOL JSM-6610LV scanning electron microscope equipped with the energy dispersive x-ray spectrometer at an accelerating voltage of 10 kV. To avoid damaging the coating, the coated samples were cut with a slow-cutting diamond wheel and epoxy was used to mount them in cross-section. Later, the surface was ground with 600 grit silica carbide paper and polished using 1 µm diamond paste. In order to reduce charge build-up and improve the samples’ surface conductivity during the SEM examination, a gold coater was utilized to sputter a thin layer of gold on the samples’ surface in an argon atmosphere. Agilent 4500 series atomic force microscope (AFM) operated in a contact mode was also utilized to analyze the coatings’ topography. The Rigaku Ultima IV x-ray diffractometer equipped with Ni-filtered Cu Kα radiation (λ = 0.154 nm) operated at 40 kV and 40 mA was utilized to assess the coatings phase composition from a range of 30 to 80° 2θ diffraction angle. The Scherrer equation was used to measure the average crystallite size.

$$D= \frac{k\lambda }{\beta cos \theta}$$

where k is a constant (0.89), λ is x-ray wavelength (0.154 nm), β is peak broadening at full width half maximum (FWHM) in radians, and θ is the Bragg's diffraction angle.

### Nanoindentation

The tests were carried out using CSM nanoindentation tester having a load range 0.5 to 300 mN. In order to ensure for limited volume penetration, a sharp three-faced pyramid Berkovich diamond tip was used. The tip of the nanoindentation tester was driven into the material at a constant load and speed for each nanoindentation. At an unload-load speed of 20 mN/min, indentations were done at loads of 10 and 20 mN. The loading and unloading cycles were separated by 60 s. The curve of load–displacement for each nanoindentation cycle was constructed by continuously monitoring the applied normal load and the displacement of the indenter tip. Hardness was evaluated using the indent’s area, which was obtained using defined geometry and tip size. For determining elastic modulus and hardness, various analytical models have been developed, including the Joslin-Oliver technique^79,80^, deformation energy technique^81,82^, the Oliver and Pharr technique^83,84^, the energy density technique^85^, and the force indentation function technique^86–88^. The Oliver and Pharr technique, used in this study, is the most popular of all the models.

The resistance to permanent deformation under a perpendicular stress is known as the nanoindentation hardness (H), and it is calculated as follows:

$$H=\frac{{P}_{max}}{{A}_{p}} \mathrm{Pascal}.$$

where P_max_ is the maximum perpendicular load, A_p_ is the projected contact area.

The elastic modulus (E) was computed from:

$$E= \frac{1- {v}_{s}^{2}}{\frac{1}{{E}_{\tau }}- \frac{1- {V}_{i}^{2}}{{E}_{i}}}$$

where E_i_ is the tip modulus, E_τ_ is the indentation contact’s reduced modulus, υ_i_ is the tip Poisson’s ratio and υ_s_ is the test sample Poisson’s ratio.

## Results and discussion

### Coatings synthesis

**Figure 1:**
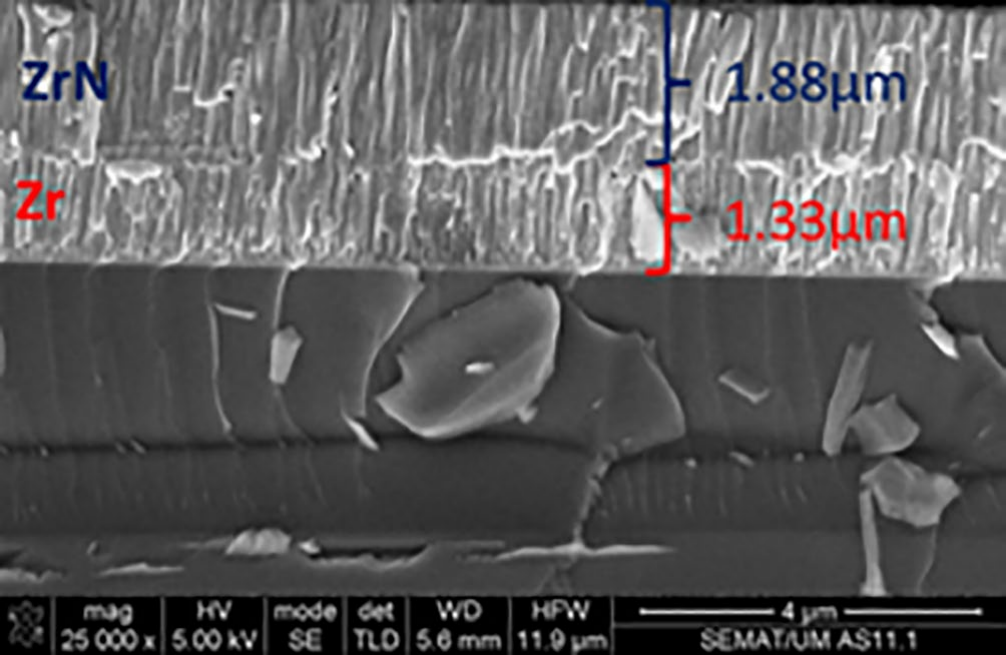
FESEM cross-section image of Zr/ZrN bilayer using a current density of 1.8 A with 0 and 11 sccm of nitrogen for Zr and ZrN.

**Figure 2:**
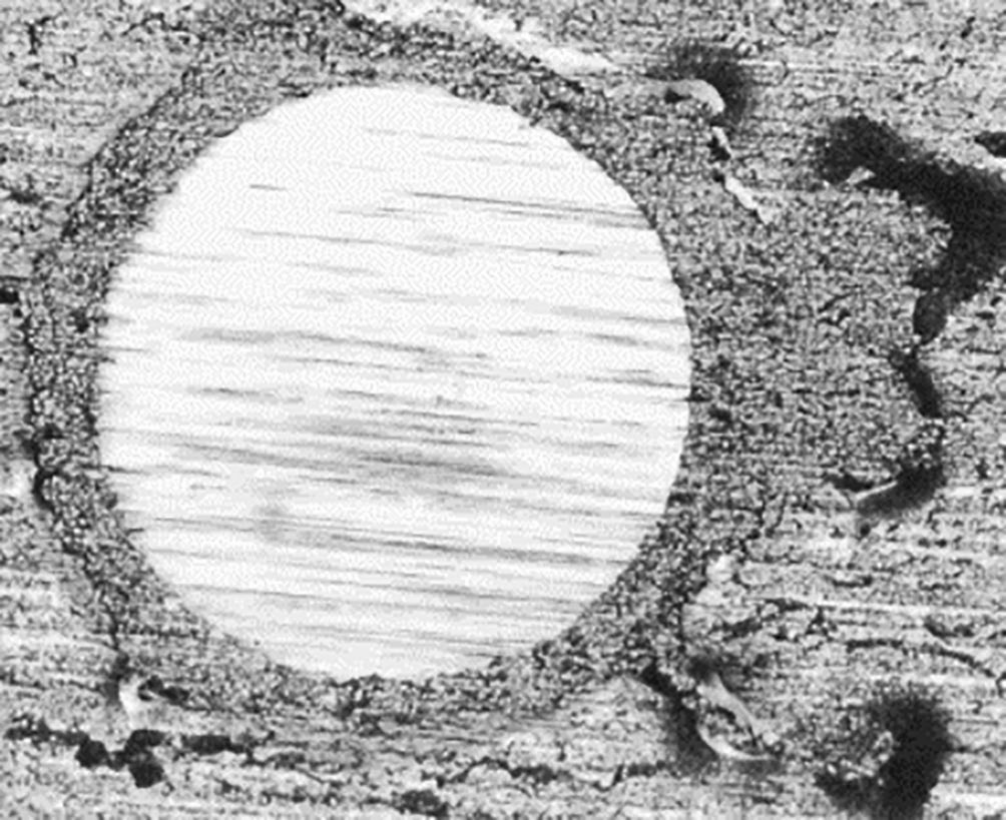
Calotest image of poorly adhered coating.

To vary the amount of N & C-atoms in the coatings and identify the rate of deposition, the optimum composition, and evaluate the cross section morphology, appropriate parameters for creating Zr, ZrN, and ZrCN coatings were selected using thick multilayer films with various N_2_ and C_2_H_2_ flows (Table 2). To determine the optimal synthesis parameters for achieving the best coating composition, thickness, and adhesion, the depositions were conducted in three series. The settings for depositing the 2nd and 3rd series of samples were chosen on the basis of the 1st series' optimization, with minor changes due to the usage of a new zirconium target, an excess of zirconium was detected. As a result, the zirconium current density was reduced and, in certain cases, gas fluxes were raised to obtain a thickness and composition identical to the first sample series. In order to alter the deposition conditions, Fig. 1 depicts the composition and thickness of two films deposited previously to the multilayer system. The target potential increased as gas fluxes increased; this increase is explained by the target surface's high reactivity with N_2_ and C_2_H_2_ gases thereby poisoning surface of the target during reactive magnetron sputtering. The target potential of multilayer coatings varied, based on how much of gas is supplied to the reactor (Table 1). To obtain the requisite wear, the monolayers’ thickness was measured utilizing the Calotest method, which involved spinning a 20 mm diameter sphere at 900 rpm for 120 s. Table 3 shows the results, whereas Fig. 1 shows a typical image for each composition. This test also provides a quantitative way to assess the films' adherence to the substrates. As, a result, coatings that failed the test were removed, and deposition conditions were optimized to increase film adherence. Figure 2 illustrates an instance of failed film during the test. To increase adhesion, a zirconium interlayer was placed between the functional layer and the substrate for each coating composition, with thicknesses ranging from 250 to 300 nm.

**Table 2:** Deposition conditions, EDS composition and deposition rates of the multilayer films used to determine the films deposition parameters.

N_2_ (sccm)	C_2_H_2_ (sccm)	Zr (At. %)	N (At. %)	C (At. %)	O (At. %)	Deposition rate (µm h^−1^)
6	3.2	51.2 ± 1.9	25.1 ± 1.2	18.4 ± 0.7	5.3 ± 0.1	3.9
6	2.8	55.6 ± 3.9	23.6 ± 3.0	15.7 ± 0.3	5.1 ± 0.7	3.2
6	2.4	59.3 ± 2.3	21.1 ± 2.3	14.8 ± 0.0	4.7 ± 0.6	3.3
10	0	48.6 ± 5.1	43.2 ± 4.1	0.0 ± 0.0	8.2 ± 1.1	1.1
9	0	46.3 + 1.5	44.9 ± 0.7	0.0 ± 0.0	8.8 ± 1.0	1.3
8	0	50.2 ± 1.9	40.5 ± 1.5	0.0 ± 0.0	9.3 ± 0.5	1.6

**Table 3:** Films thicknesses, grain size and deposition rates.

Sample ID	Coating composition	Individual Layer thickness (nm)	Ø C_2_H_2_ (sccm)	Ø N_2_ (sccm)	Time (s)	Grain size (nm)	Coating thickness (µm)	Deposition rate (µm h^−1^)
PN7	Zr/ZrN	500	0	0–22	2940	12.6	1.96 ± 0.14	2.4 ± 0.2
PN8	Zr/ZrN	500	0	0–22	4410	12.7	3.22 ± 0.20	2.6 ± 0.2
PN9	Zr/ZrN	250	0	0–22	2940	8.50	2.12*	
PN10	Zr/ZrN	250	0	0–22	4410	9.25	2.96 ± 0.03	2.4 ± 0.0
PN11	ZrN/ZrCN	500	0–16	22–12	3024	5.96	2.02*	
PN12	ZrN/ZrCN	500	0–16	22–12	4536	5.55	3.00 ± 0.06	2.4 ± 0.1
PN13	ZrN/ZrCN	250	0–16	22–12	3024	8.50	2.02*	
PN14	ZrN/ZrCN	250	0–16	22–12	4536	9.25	2.99 ± 0.01	2.4 ± 0.0

*Thicknesses and standard deviation are estimated using deposition rates calculated by means of calotest and SEM images.

**Figure 3:**
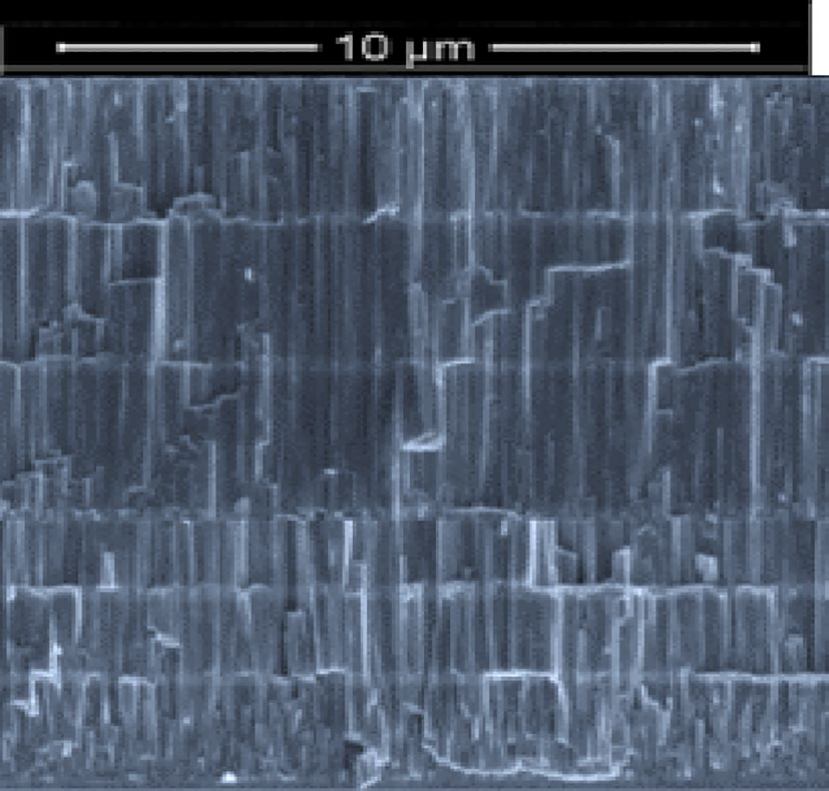
FESEM cross-section images of the deposited films.

**Figure 4:**
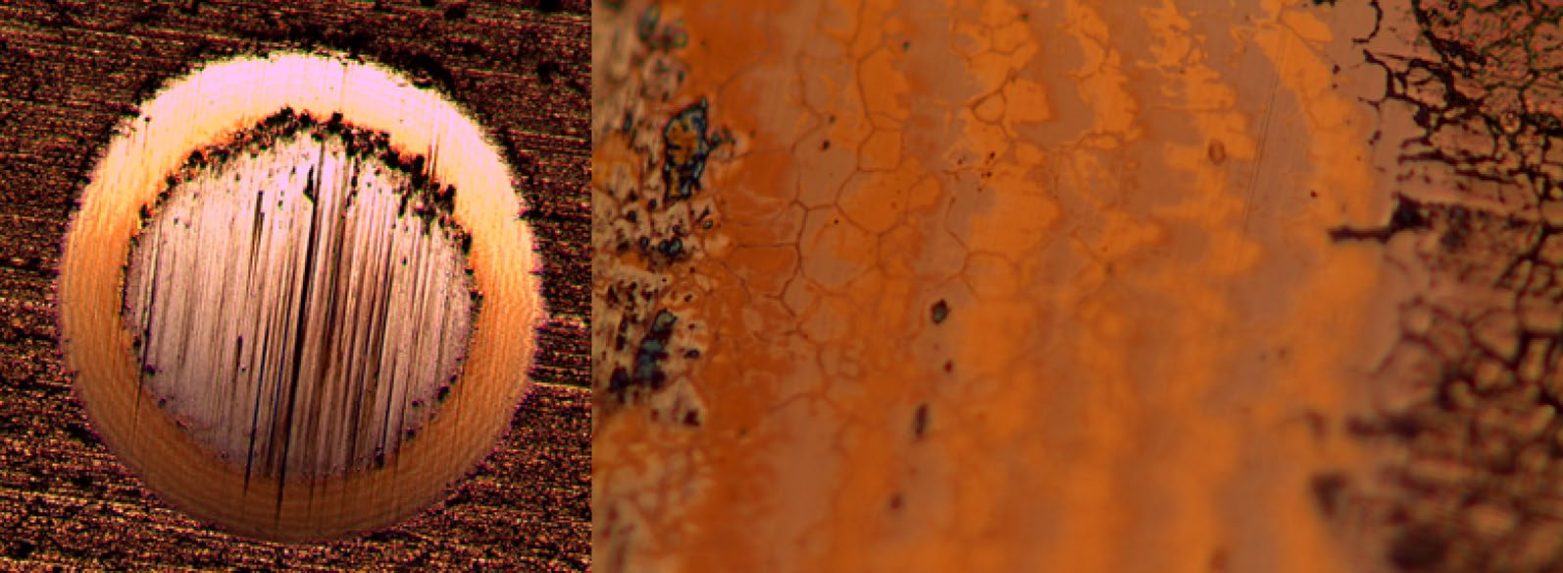
Calotest images for (left image) PN14 at ×5 and (right image) PN14 at ×50.

The multilayer coating thicknesses were optimized making use of the monolayer thickness and SEM images in order to determine the deposition conditions of each compound. The coatings' cross-sectional FESEM views mostly exhibited columnar structures with a few small grains at the coating/alloy interface (Fig. 3). Such structure is distinctive of sputter-deposited coatings, which are formed at high super-saturation conditions, resulting in high rate of nucleation with tiny nuclei at the base^89,90^. The primary columnar shape found in coatings explored here is a consequence of motion of grain boundary in the course of the epitaxial and coalescence developments. The Thornton model^91^, which proposes that faults from atomic shadowing & voided growths contribute to limited sample’s surface diffusion, supports this type of growth. The columnar morphology for zirconium-based coatings have been documented extensively^14^. ZrN is a dazzling gold, whereas ZrCN is a dull gold and pure Zr coating is silver grey in color. This is consistent with previous findings of silver-colored metallic coatings containing high Zr and low nitrogen^92,93^. The creation of stoichiometric ZrN, which has a gold color, occurs when the nitrogen content in the coatings rises^44,92,93^. Additionally, Calotest was used to characterize the thickness of the multilayer where possible and the results are presented in Table 3. It must be stressed that Calotest allows the observation of the multilayer, as observed in Fig. 4, however the thickness of each individual layer was not calculated since the error of the technique may exceed the thickness of the individual layers. Figure 4 presents representative images of the multilayer for 250 of thickness of each individual layer. When nitrogen is added to the process, the film deposition rate decreases, as expected due to Zr targets’ poisoning; however, when acetylene is used instead, the deposition rate increases, this is explained by carbon's ability to deposit directly into the films.

**Figure 5:**
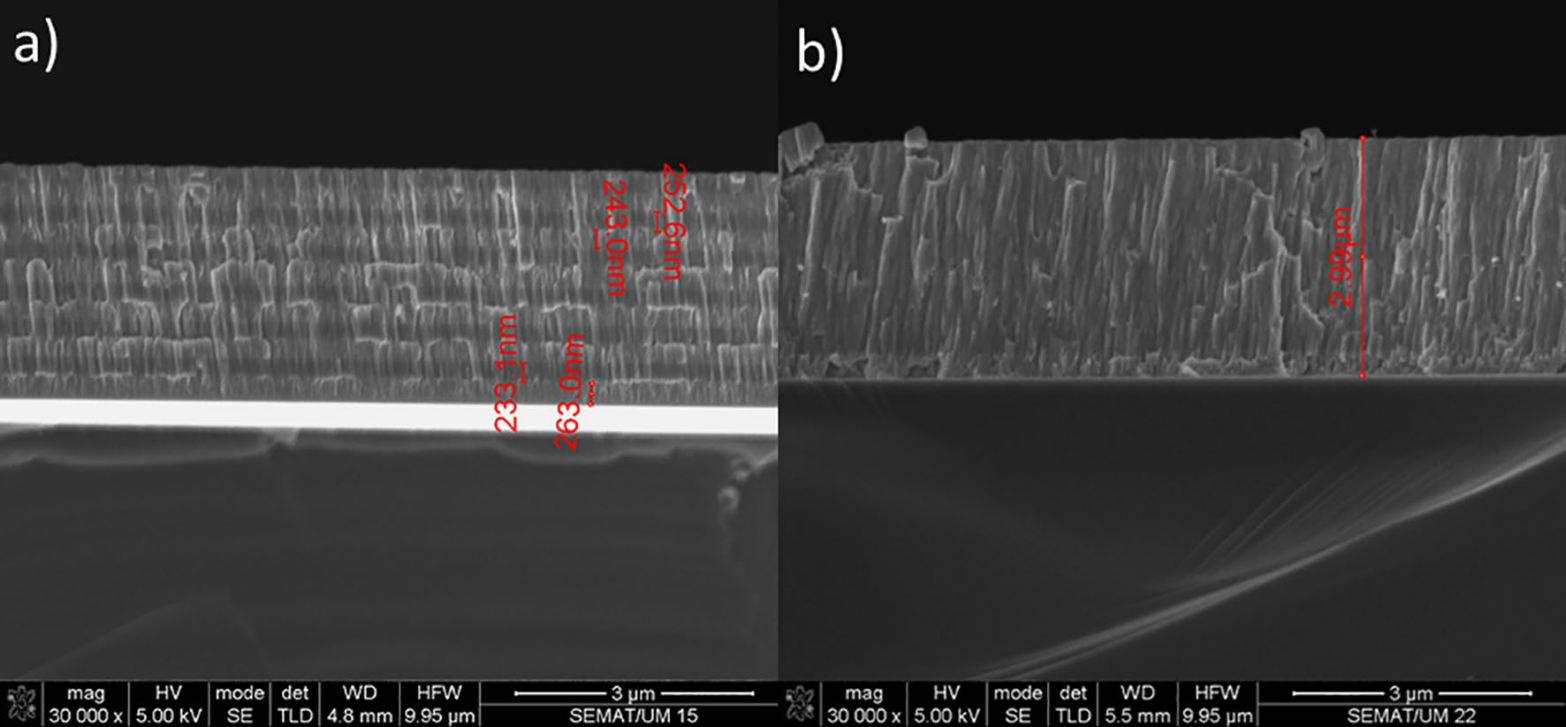
Cross-section FESEM images for samples (**a**) PN10 and (**b**) PN14.

Multilayer cross-sectional SEM images are presented in Fig. 5 for two representative samples PN10 and PN14, corresponding to Zr/ZrN and ZrN/ZrCN multilayer, respectively. Figure 5a reveals a clear differentiation between the Zr and ZrN layer, with individual layer thicknesses close to 250 nm. This individual layer is not clearly noticed for the ZrN/ZrCN films, since structural and morphological changes for those materials are expected to be very subtle; nonetheless, the layer differentiation was detected by optical images after Calotest, as previously discussed. In contrast, Zr and ZrN may present different crystalline phases and the large compositional/morphology variation, as observed for surface SEM images and hence, each layer is clearly identified.

### Materials characterization

#### SEM/EDS

**Figure 6:**
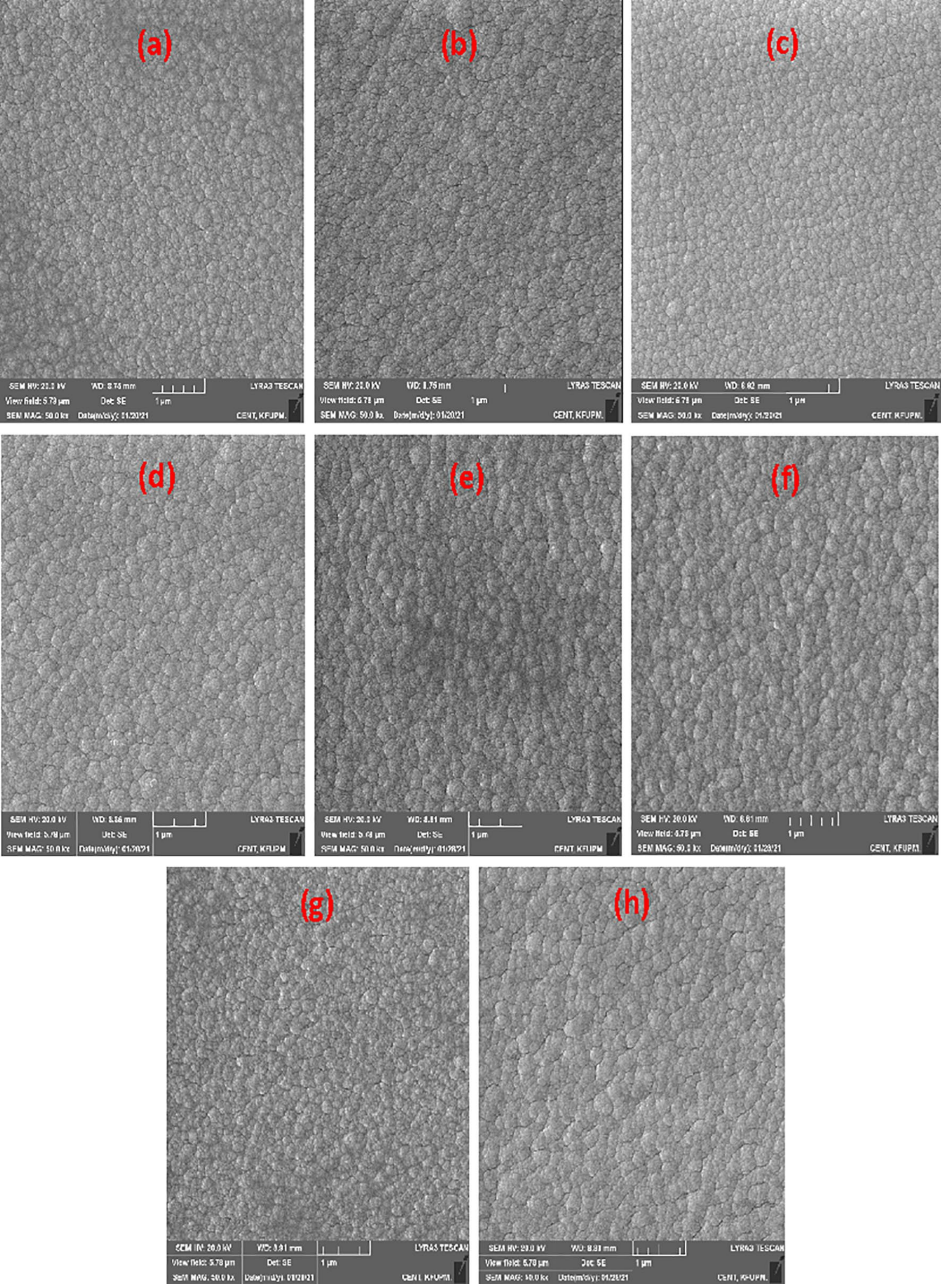
High magnification FESEM micrographs of (**a**) PN7, (**b**) PN8, (**c**) PN9, (**d**) P10, (**e**) PN11, (**f**) PN12, (**g**) PN13 and (**h**) PN14 coatings’ top surfaces.

The morphology of the coatings' top surfaces was studied using FESEM. The FESEM secondary electron micrographs of the coatings are shown in Fig. 6. The morphology of the coatings was essentially homogeneous, compact and dense with fine spherical grains. This is consistent with the previously observed columnar grain growth (Fig. 1). Aside from usual grain growth, localized grain growth near the thickening film's surface was seen, which was aided by nearby grain coalescence. Figure 6 demonstrates that for all coating compositions, coatings with a 3 µm thickness had larger grain sizes than those with a 2 µm thickness. The larger grain growth in the 3 µm coatings is attributed to the prolonged time of deposition, which may have allowed adjacent atoms to reorganize via surface diffusion, resulting in bigger grains. Generally, the grains of the ZrN/ZrCN coatings were more compact and smaller compared to those observed in the Zr/ZrN coatings. It has been reported that the carbon and nitrogen atoms in coatings that contain these atoms tend to limit grain growth by reducing neighboring grains’ mobility, resulting in fine grain size^78^, thus, resulting in the smaller grain size of the ZrN/ZrCN as compared to the Zr/ZrN coatings observed in this study. This is because carbon and nitrogen limit the mobility of adjacent grains and restrict them to the fine grains. Furthermore, densification of coatings was observed in the present study due to introduction of carbon and nitrogen. This leads to filling of interstitial positions due to diffusion of carbon and nitrogen which reduces the vacant cites, which in turn leads to more dense and smooth morphology of the coatings^93^.

**Figure 7:**
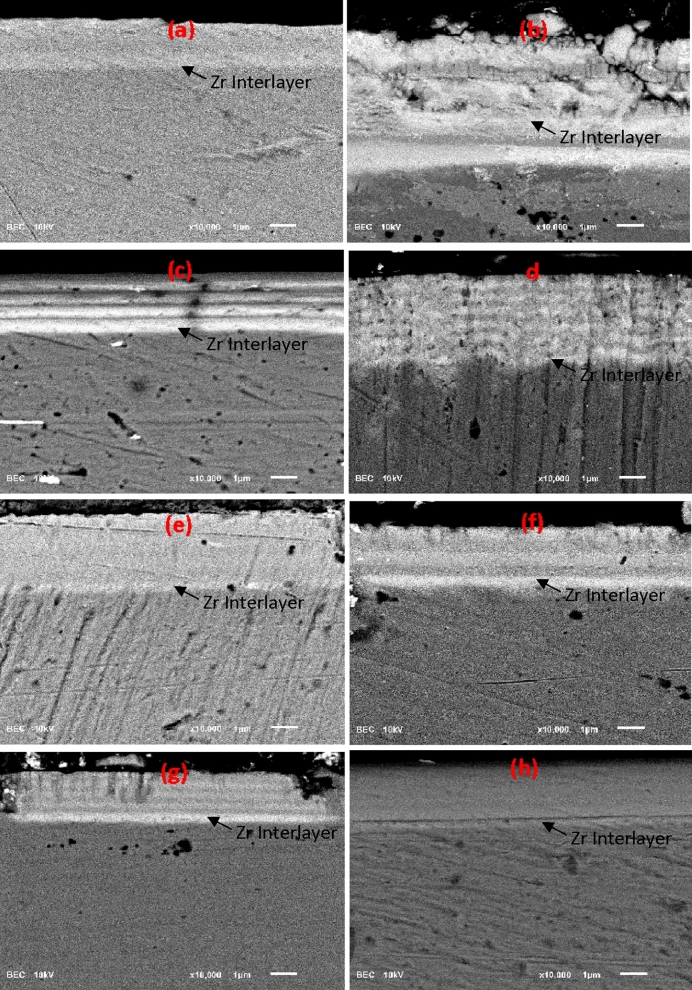
Backscattered electron SEM micrographs of **(a)** PN7, **(b)** PN8, **(c)** PN9, **(d)** P10, **(e)** PN11, **(f)** PN12, **(g)** PN13 and **(h)** PN14 coatings’ cross-section.

The cross-sectional backscattered electron image (BEI) of all coatings are shown in Fig. 7. It's clear that the deposition parameters used were successful in achieving the intended number of layers and thickness for each coating. The individual layers for each coating are of equal thickness. The Zr interlayer between the metal substrate and the functional layers is clearly visible (the first bright color) layer in all the coatings. The thickness of the Zr interlayer was 250 nm to 300 nm. The reason to insert Zr interlayer between substrate and functional layer is to improve their adhesion. The Zr interlayer is marked in Fig. 7. The coated samples exhibit well-defined multilayer structure with good planarity of individual layers and distinctive interfaces. For the Zr/ZrN multilayer coating, the alternating bright and grey color regions represent the Zr and ZrN layers respectively (Fig. 7a–d). While for the ZrN/ZrCN multilayer coatings, the alternating grey and dark color regions represent the ZrN and ZrCN layers respectively (Fig. 7e–h).

**Figure 8:**
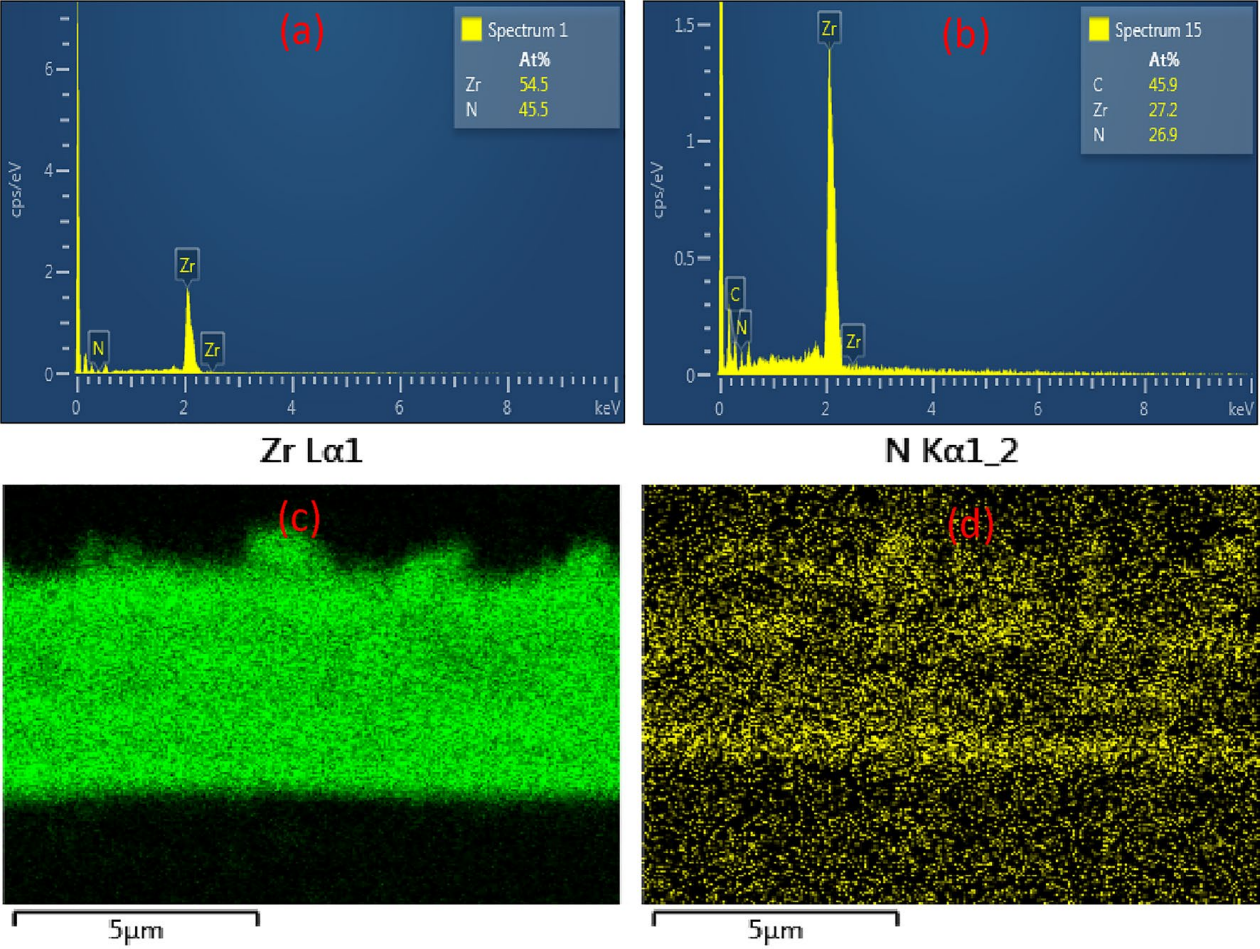
EDS spectra of **(a)** PN8 & **(b)** PN12 and X-ray maps of **(c)** Zr and **(d)** N for PN8.

Figure 8 depicts representative cross-sectional EDS spectra for PN8 (Fig. 8a) & PN12 (Fig. 8b) and x-ray mapping for PN8 (Fig. 8c,d). EDS spectrum of PN8, whose result is representative of all the Zr/ZrN coatings, reveals a higher atomic mass % for Zr as compared to N with N to Zr ratio 0.835 (Fig. 8a). This is expected due to the presence of single Zr layers with the coatings. The error in the Zr and N was observed as 54.5 ± 1.85 and 45.5 ± 1.85, respectively. The ZrN/ZrCN coatings, represented by the result for PN12 coating, exhibited an almost stoichiometric N to Zr ratio of 0.989 with the carbon atomic % almost twice the atomic % for either Zr or N (Fig. 8b). The error in the C, Zr and N was noted as 45.9 ± 2.38, 27.2 ± 2.68, and 26.9 ± 1.99, respectively. The representative x-ray mapping also shows the alternating multilayer structure of the coatings, the individual Zr and ZrN coatings for coating PN8 are clearly depicted (Fig. 8c,d).

#### XRD analysis

**Figure 9:**
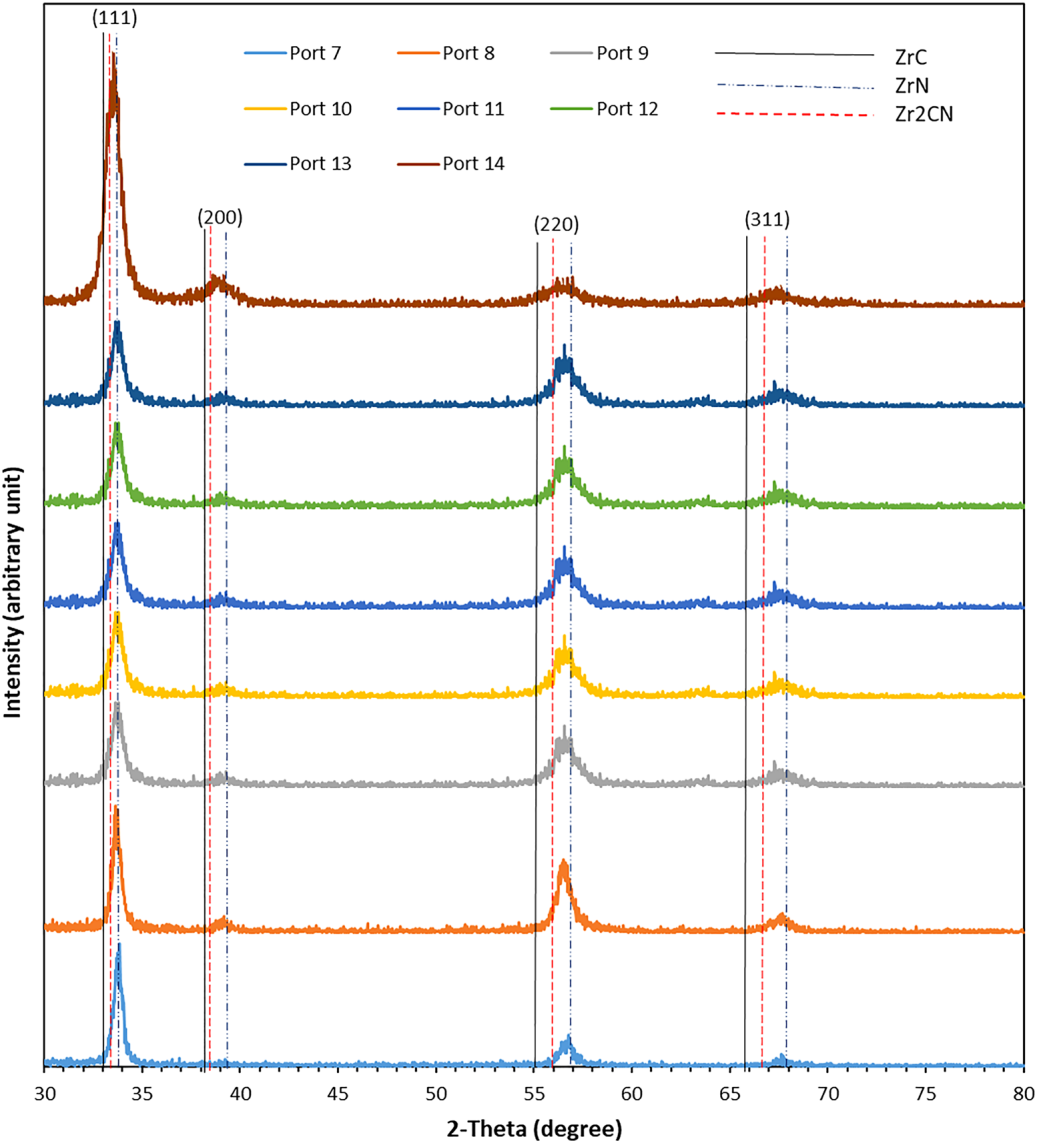
Obtained XRD spectra obtained for **(a)** PN7, **(b)** PN8, **(c)** PN9, **(d)** P10, **(e)** PN11, **(f)** PN12, **(g)** PN13, and **(h)** PN14 coatings (the Zr, ZrN and Zr_2_CN coatings compositions are identified).

ZrN is a refractory ceramic substance with a high melting point. It is covalently bonded and has fcc cubic structure. ZrCN forms a quasi-binary solid solution when C and N atoms occupy the octahedral interstitial sites^79^. Figure 9 shows the x-ray diffraction spectra obtained from all the multilayer coatings. The spectra match that of an analogous coating described in a recent study^94,95^, with obvious (fcc) ZrC, Zr_2_CN and ZrN, peaks that are almost homogeneous. The standard peak positions of (fcc)-ZrC (1CCD: 00-035-0784), (fcc)-Zr_2_CN (ICSD: 01-071-6065) and (fcc)-ZrN (ICCD: 00-035-0753) are plotted as dashed lines. All the coatings peaks were in agreement with the standard ZrN and Zr_2_CN. All the coatings exhibited preference for (111) plane. The (220) planes also exhibited a high peak. The (111) plane have been reported as the preferred orientations for ZrN and Zr_2_CN coatings^17,21,38,78^. Other planes that showed low-intensity peaks were planes (200) and (311).

The ZrN/ZrCN coatings had smaller grain size than the Zr/ZrN coatings, this corresponds to the surface morphology of the coatings as observed in the FESEM micrographs (Fig. 6). This is because the carbon atoms in coatings limit grain growth, resulting in tiny grain size. Also in general, the coatings of 3 µm thickness had larger grain size than the ones in 2 µm thickness, this is a consequence of the prolonged deposition times for the coatings of 3 µm thickness as against the 2 µm ones (Table 3).

#### AFM analysis

**Figure 10:**
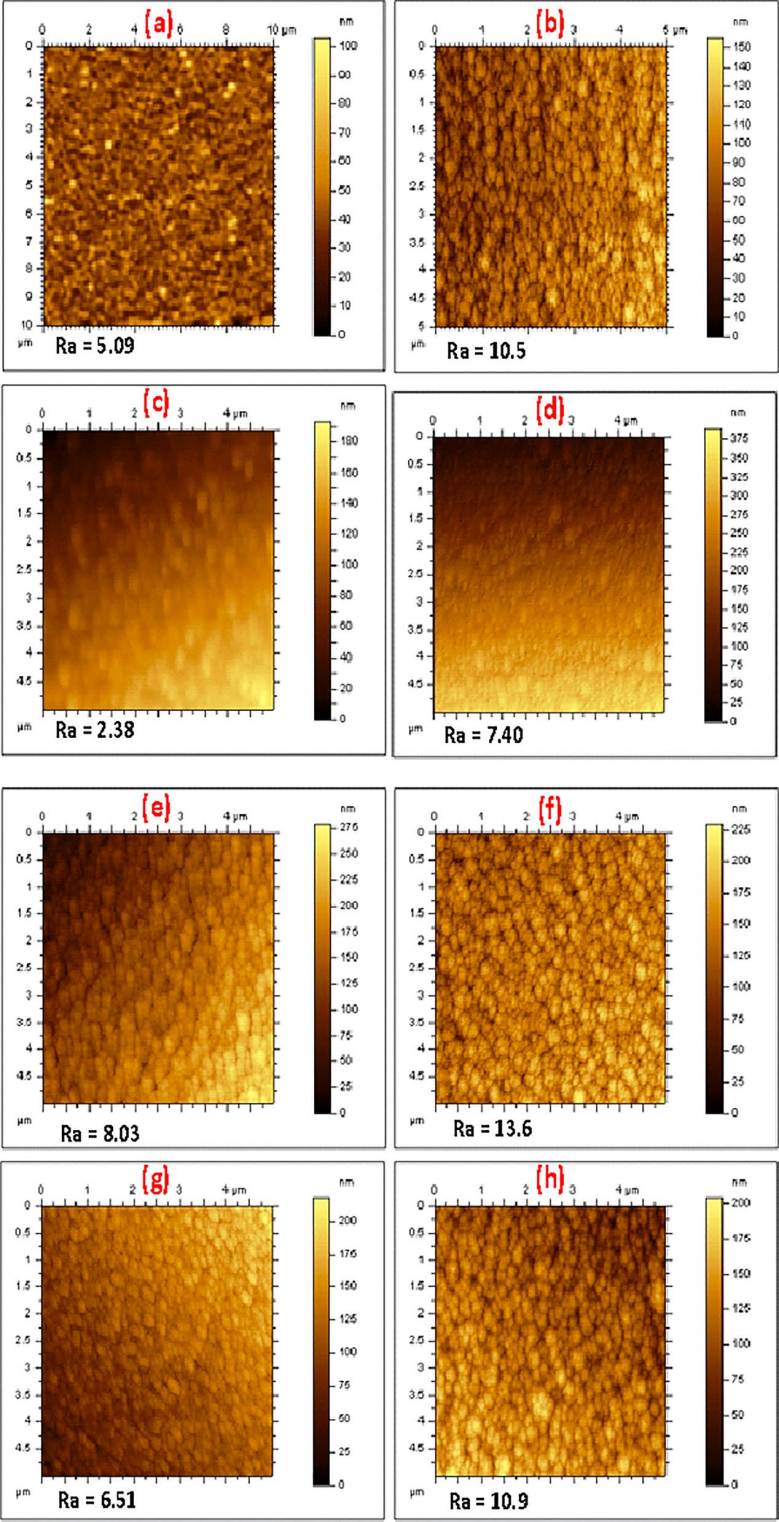
2D AFM images obtained from the top surfaces (5 × 5 µm area) of **(a)** PN7, **(b)** PN8, **(c)** PN9, **(d)** PN10, **(e)** PN11, **(f)** PN12, **(g)** PN13, **(h)** PN14 multilayer coatings. The quoted Ra value for each coating is the average of Ra values obtained at 11 different points across the 5 × 5 µm area.

The 2D AFM images of all the coatings are shown in Fig. 10. Just as it was observed in the SEM, all the coatings showed compact, uniform and dense morphology of fine spherical grains with the 3 µm coatings appearing more compact, coarse and having grains of larger size than the 2 µm coatings. Also, the 3 µm coatings have larger average roughness (Ra) value than the 2 µm coatings. The larger average roughness value of the 3 µm coatings as against the 2 µm coatings is a consequence of increased clustering and packing which leads to increase vertical size of grains^96^.

### Nanoindentation

**Figure 11:**
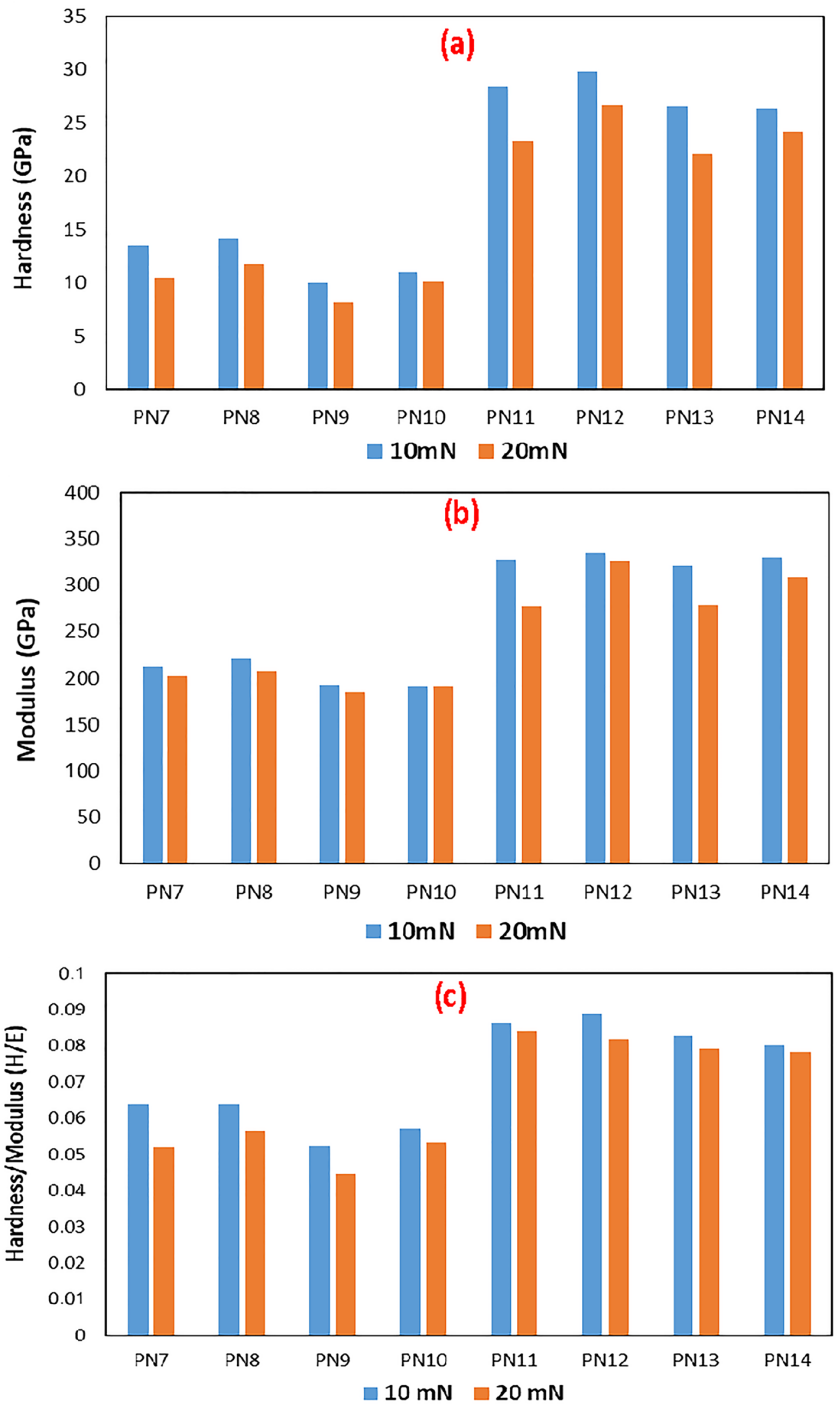
Bar charts showing values of **(a)** hardness, **(b)** elastic modulus and **(c)** hardness/elastic modulus (H/E) values for all the studied multilayer coatings.

All nanoindentation tests of all coatings were performed at two different loads (10 & 20 mN). After which, the load (change in applied normal force) vs displacement (depth of penetration) of the indenter over time was plotted. For the same indent, the normal force was plotted vs penetration depth. For all coating compositions, depth of penetration increases with load, ranging from 145 nm (for ZrN/ZrCN) to 435 nm (for Zr/ZrN). The indenter penetration depth for ZrN/ZrCN coatings was within the ideal range (10% of coating thickness). Whereas the Zr/ZrN coatings have penetration depths ranging from 11 to 20% of the coating thickness. This is owing to the fact that Zr/ZrN coatings have lower hardness. The Oliver & Pharr (O & P) method was used to calculate the elastic modulus and nanoindentation hardness from the plots^62,63^. Figure 11 shows bar charts of nanohardness and elastic modulus plotted for each coating mixture at loads of 10 and 20 mN.

Hardness is controlled by many factors such as preferred orientation, residual stress, grain size, defect density, stoichiometry and structure of the coatings which is turn depend largely on film growth conditions and process parameters including the synthesis technique, substrate bias, growth temperature, etc.^97^ Fig. 11a shows that the Zr/ZrN multilayer coatings generally have lower hardness values as compared to the ZrN/ZrCN coatings where sample PN12 (3 µm, ZrN/ZrCN multilayer coating having individual layer of each 500 nm in thickness) exhibited the highest hardness of 29.82 and 26.62 GPa at loads of 10 and 20 mN, respectively. Whereas, the 2 µm Zr/ZrN multilayer coating with individual layer thickness 250 nm (sample PN9) exhibited the least hardness of 9.99 and 8.18 GPa at loads of 10 and 20 mN, respectively. The presence of carbon in the ZrN/ZrCN coatings makes the ZrN/ZrCN coatings denser and compact as observed in the FESEM images (Fig. 6). It has been reported the denser and more compact a coating the harder it is^1,98,99^. As explained above the introduction of carbon leads to dense, firm, smooth and small grain size morphology. High hardness values are achieved for highly dense and nanocrystalline coatings. This is because a highly dense nanostructured coating inhibits sliding or rotation of crystallites due to the lack of free space, thus giving rise to high hardness values^97^. Also for both multilayer coating types, the hardness decreases with decrease in bilayer thickness (Fig. 11a). The influence of the substrate on hardness measurements is reduced by keeping the indent displacement to 10% of the coating thickness. In contrast, a small indent could be comparable in size to the sample surface roughness, reducing measurement accuracy. As a result, there will be a discrepancy in the measurement results. To avoid this, the penetration depth must be at least 20 times the roughness of the surface. The majority of the time, a compromise between these two competing conditions is reached. The change from an elastic to an elastic/plastic state in the indented region makes measurement difficult at low stresses^62^. Soft films on hard surfaces typically increase in hardness as the indenter penetration depth increases, whereas hard films on soft substrates frequently decrease in hardness as displacement increases. For all the coatings, the hardness value was observed to decrease with increase in load from 10 to 20 mN. Stainless steel substrate influence on coating hardness at both load 10 and 20 mN has been discussed in a previous study^78^. It was reported there that the hardness of the substrate was measured as 2.10 GPa. The substrate was reported to have little effect on the coatings hardness at 10 mN while at the 20 mN load, the substrate showed a more significant effect on the coating hardness, it reduced the coating hardness at this load. Also the effect of substrate on coating hardness was also reported to reduce with coating thickness^78^. All these suggest that the underlying stainless steel substrate possessed a much lesser hardness than the coatings.

Similar trends just as in the case of hardness was observed for elastic modulus. The Zr/ZrN multilayer coatings generally have lower values of elastic modulus as compared to the ZrN/ZrCN coatings where sample PN12 (3 µm, ZrN/ZrCN multilayer coating having individual layer each 500 nm in thickness) exhibited the highest elastic modulus of 335.3 and 325.2 GPa at load 10 and 20 mN respectively. Whereas, the 2 µm Zr/ZrN multilayer coating with individual layer thickness, 250 nm (sample PN9) showed the lowest elastic modulus of 190.8 and 184.0 GPa at load 10 and 20 mN respectively (Fig. 11b).

It is well understood that hardness is not the only criterion for predicting coating wear, the ratio of hardness to modulus (H/E) which is related to elastic strain to failure and fracture toughness has been proposed as one of the key parameters controlling wear^100–102^. In fact, the H/E ratio has been shown by some authors to be a more appropriate parameter for predicting wear resistance compared to hardness, which can be regarded as a vital material property that defines wear resistance^101,102^. Similar to hardness and modulus, the H/E ratio of ZrN/ZrCN multilayer coatings was higher than that of Zr/ZrN coatings, with PN12 coatings showing the highest H/E value and PN9 coatings having the lowest H/E value (Fig. 11c). A high value of H/E suggests a higher fracture toughness. This implies that the ZrN/ZrCN multilayer coatings generally showed higher fracture toughness than the Zr/ZrN multilayer coatings. The higher hardness and H/E ratio of the ZrN/ZrCN coatings as compared to the Zr/ZrN coatings is due to the presence of carbon in the ZrN/ZrCN coatings as explained earlier.

## Conclusions

Using a dc magnetron sputtering method and varied N_2_ and C_2_H_2_ flowrates so as to manipulate the coatings nitrogen and carbon contents, Zr/ZrN and ZrN/ZrCN multilayer coatings, each with individual layer thicknesses of 250 and 500 nm and coatings thicknesses of 2 and 3 µm were deposited on 316L stainless steel. The coatings morphology and microstructure were investigated by high resolution FESEM, BEI, EDS, XRD and AFM techniques. Nanoindentation method was employed to measure the hardness and modulus of the coatings. The ratio of hardness to modulus (H/E) which is related to elastic strain to failure was also employed in the study. The cross-section FESEM images of the coatings reveal the coatings to show mainly growth that is columnar in nature with some tiny grains at the substrate/coating interface. The top surface FESEM images show a homogeneous, compact and dense morphology of fine spherical grains for all the coatings with the ZrN/ZrCN coatings showing more compact and smaller grain size than the Zr/ZrN coatings. The BEIs show alternating bilayers of equal sizes for each coatings. The denser and more compact nature of the ZrN/ZrCN as compared to the Zr/ZrN coatings due to the carbon presence in ZrN/ZrCN resulted in higher hardness, modulus and H/E exhibited by the ZrN/ZrCN coatings. This suggests the ZrN/ZrCN coatings exhibit a higher wear resistance and fracture toughness than the Zr/ZrN coatings. The effective synthesis and excellent characteristics shown by the studied multilayer coatings make them viable candidates for tribological applications.
